# Improved joint line and posterior offset restoration in primary total knee replacement using a robotic-assisted surgical technique: An international multi-centre retrospective analysis of matched cohorts

**DOI:** 10.1371/journal.pone.0272722

**Published:** 2022-08-25

**Authors:** Ravi Popat, Ali Albelooshi, Piyush Mahapatra, Peter Bollars, Max Ettinger, Simon Jennings, Jan-Louis Van den Berg, Dinesh Nathwani

**Affiliations:** 1 Imperial College London, London, United Kingdom; 2 Mediclinic City Hospital, Dubai, UAE; 3 London North West University Healthcare NHS Trust, London, United Kingdom; 4 St Trudo Hospital, Sint Truiden, Belgium; 5 Hannover Medical School, Annastift Hospital, Hannover, Germany; 6 Busmaed Paardevlei Private Hospital, Cape Town, South Africa; Paracelsus Medizinische Privatuniversitat - Nurnberg, GERMANY

## Abstract

**Background:**

Accurate restoration of joint line height and posterior offset in primary Total Knee Arthroplasty (TKA) have been shown to be important factors in post-operative range of movement and function. The aim of this study was to assess the accuracy of joint line and posterior offset restoration in a group of patients that underwent robotic-assisted TKA (raTKA). A matched cohort of patients that underwent a TKA using a conventional jig-based technique was assessed for comparison. The null hypothesis was that there would be no difference between groups.

**Methods:**

This study was a retrospective analysis of a cohort of 120 patients with end-stage knee osteoarthritis that received a TKA using the Navio Surgical System (n = 60), or Conventional manual TKA (n = 60). Procedures were performed between 1 January 2019 and 1 October 2019 at six different centres. Joint line height and posterior offset was measured pre-operatively and post-operatively on calibrated weight bearing plain radiographs of the knee. Two observers performed measurements using validated measuring tools. A BMI and age-matched cohort of patients that underwent TKA using a conventional technique in the same six centres were assessed for comparison. Mean values, standard deviations and confidence intervals are presented for change and absolute change in joint line height and posterior offset. Student’s t-test was used to compare the changes between techniques.

**Results:**

Patients that underwent robotic-assisted TKA had joint line height and posterior offset restored more accurately than patients undergoing TKA using a conventional technique. Average change from pre-operative measurement in joint line height using raTKA was -0.38mm [95% CI: -0.79 to 0.03] vs 0.91 [0.14 to 1.68] with the conventional technique. Average absolute change in joint line height using raTKA was 1.96mm [1.74 to 2.18] vs 4.00mm [3.68 to 4.32] with the conventional technique. Average change in posterior offset using raTKA was 0.08mm [-0.40 to 0.56] vs 1.64mm [2.47 to 0.81] with the conventional technique. Average absolute change in posterior offset with raTKA was 2.19mm [1.92 to 2.46] vs 4.24mm [3.79 to 4.69] with the conventional technique. There was a significant difference when comparing absolute change in joint line height and posterior offset between groups (p<0.01).

**Conclusion:**

Robotic-assisted primary TKA restores the joint line height and posterior offset more accurately than conventional jig-based techniques.

## Introduction

Change in anatomical joint line height can have a significant impact on post-operative function following Total Knee Arthroplasty (TKA) [1–4]. Instability associated with altered joint line is most evident in mid-flexion [1]. Change in joint line height can have a significant impact on knee flexion. Ryu et al. have demonstrated that patients with better post-operative knee flexion have had better preservation of the natural joint line [5]. This effect is potentially more significant in cruciate-retaining designs [6].

Proximal shift of the joint line can result in patella baja [7], impingement of the patella and patella tendon on the tibial component, and inefficiency of the quadriceps mechanism [8]. Distal displacement of the joint line can result in pain and subluxation [9–11]. The mechanics of the patellofemoral joint is affected by change in joint line height, with contact forces demonstrated to increase by 60% if the joint line is elevated by 10mm [8].

Functional outcomes are also influenced by change in joint line height. The seminal work by Figgie et al [12] demonstrated better functional outcomes following primary TKA when there was less than 8mm of joint line elevation. Better functional scores have been demonstrated in revision surgery when the joint line has been maintained [13].

Navigation was introduced to help surgeons achieve more accurate positioning of implants. Studies have demonstrated that computer-assisted surgery improves component alignment and mechanical axis following TKA [14–18]. Previous studies have shown that the use of computer-assisted techniques does not improve a surgeon’s ability to restore the natural joint line in primary TKA [19, 20]. Computer assisted surgery has been shown to be effective in restoring the joint line in revision surgery when compared with conventional methods [21].

The primary aim of this study was to determine whether raTKA more accurately restored joint line height when compared to TKA performed using a more conventional technique. The secondary outcome was to assess whether raTKA restored posterior offset more accurately, when compared to conventional surgery. The null hypothesis was that there would be no difference between raTKA and a conventional technique when assessing change in joint line height and change in posterior offset in TKAs.

## Methodology

This study was a retrospective analysis of a cohort of patients with end-stage knee osteoarthritis. 60 patients received a TKA using the Navio Surgical System between 1 January and 1 October 2019 at six different centres in five countries (Imperial College Healthcare NHS Trust, London, UK; London North West University Healthcare NHS Trust, London, UK; Hannover Medical School, Annastift Hospital, Hannover, Germany; St Trudo Hospital, Sint Truiden, Belgium; Busmaed Paardevlei Private Hospital, Cape Town, South Africa, Mediclinic City Hospital, Dubai, UAE). A BMI and age-matched cohort of 60 patients that underwent TKA using a conventional technique in the same six centres were assessed for comparison.

**Fig 1:**
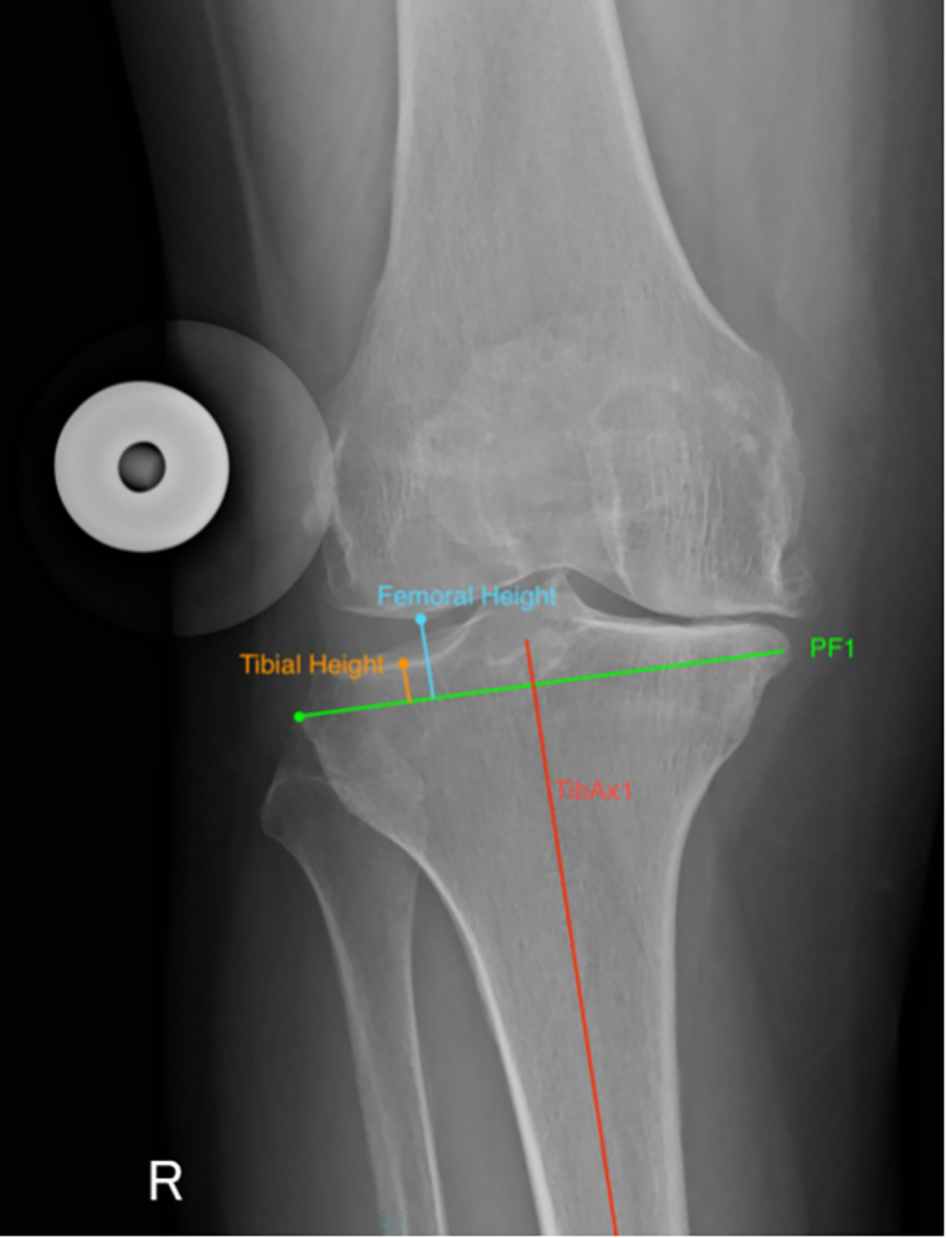
Pre-operative IJLCM technique. Assessment of pre-operative joint line height calculated as the average value of the tibial height and femoral height on the least affected side. *TibAx1 = the intramedullary axis of the tibia*, *PF1 = a line perpendicular to TibAx1 at the level of the most proximal point of the proximal fibula*.

**Fig 2:**
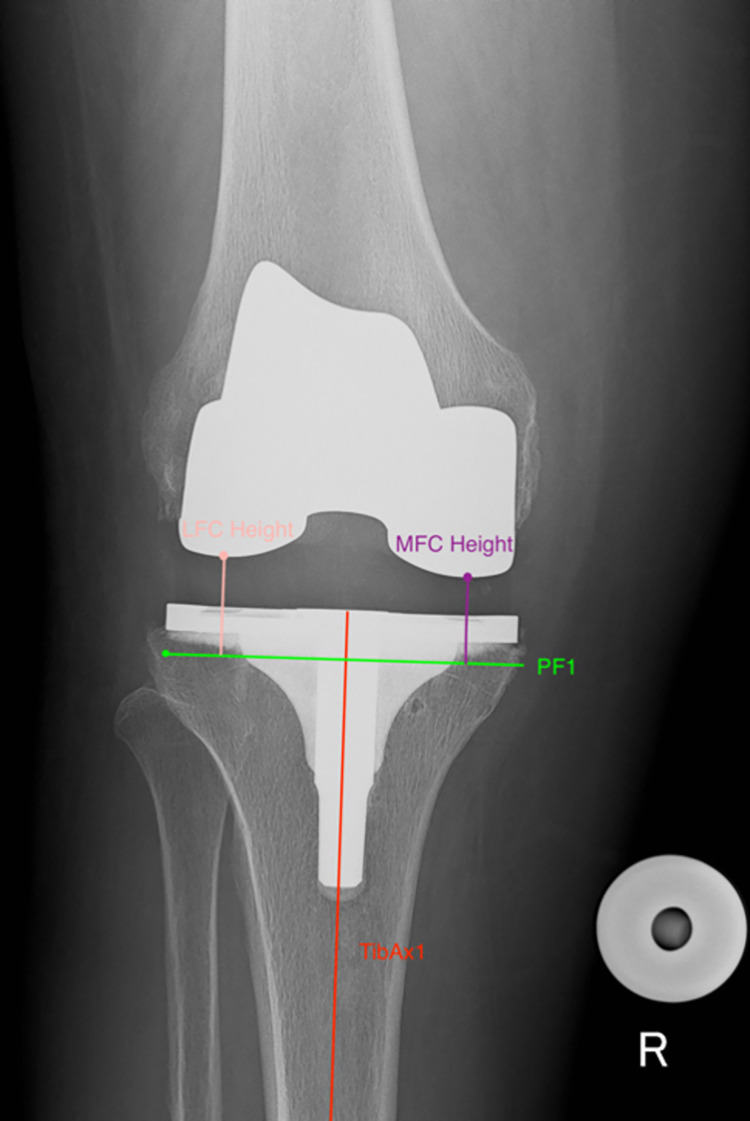
Post-operative IJLCM technique. Assessment of post-operative joint line height calculated as the average value of the Lateral Femoral Condyle (LFC) Height and the Medial Femoral Condyle (MFC) Height.

**Fig 3:**
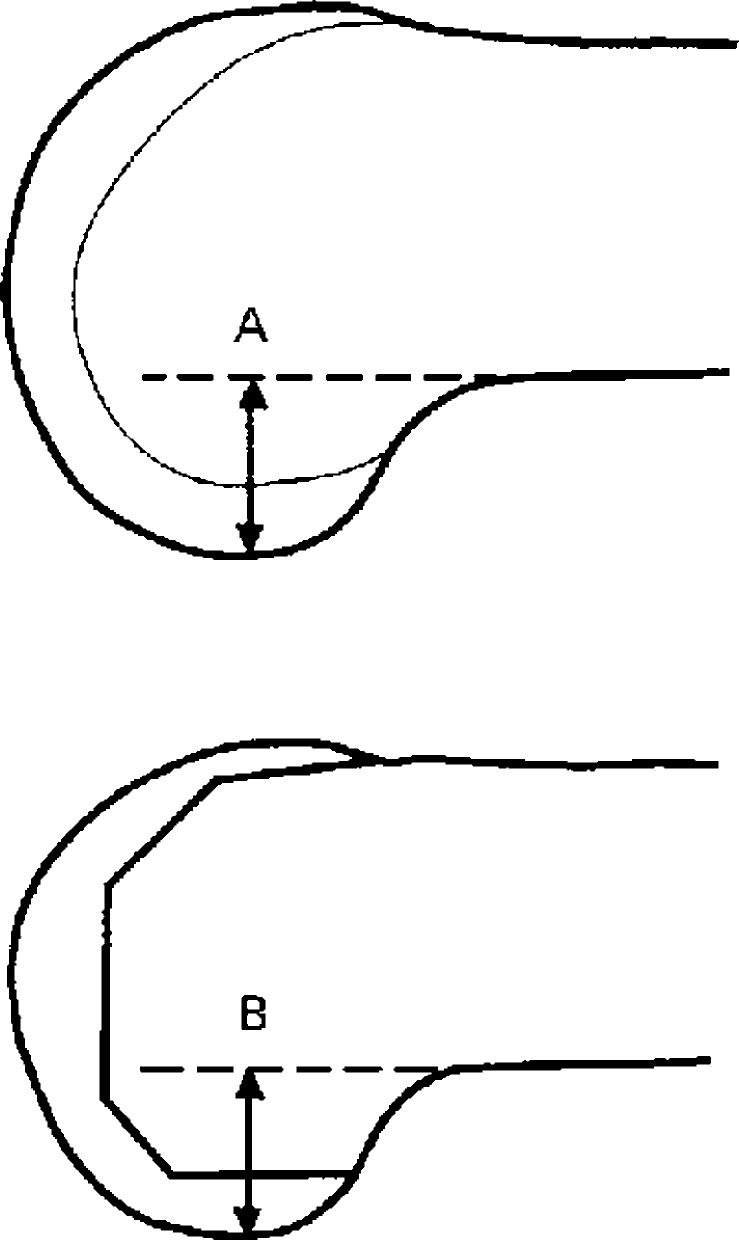
Posterior offset measurement. Diagram showing the measurement of posterior condylar offset (A) before and (B) after operation.

Pre- and post-operative images (weight bearing and calibrated antero-posterior (AP) and lateral radiographs) for all 120 patients were accessed by the authors, or members of their team retrospectively. Images were then anonymised before being sent to the observers (RP and PM). Images were accessed on 1 December 2019. Computer software Osirix (Pixmeo, Bernex, Switzerland) was used to perform measurements. Joint line height was measured on pre-operative and post-operative radiographs using the Imperial Joint Line Congruency Measurement (IJLCM) Technique [22] (Figs 1 and 2). Posterior offset was measured using the technique described by Bellemans et al [23] (Fig 3). All pre-operative and post-operative images were reviewed by two Orthopaedic Surgeons. Both observers performed two full sets of measurements separately. Each set of measurements was performed two weeks apart.

### Radiographic quality

All patients underwent weightbearing AP and lateral plain radiography of the affected knee joint before and after joint replacement. All radiographic images were reviewed by both observers to ensure adequate image quality and rotational profile. Exclusion criteria included images where accurate calibration could not be performed (using calibration disc on pre-operative radiographs and the tibial keel on post-operative radiographs), patients with a fixed flexion deformity pre- or post-operatively (as these deformities affect joint line height measurements), images where the most proximal point on the fibula was not visible and images where the longitudinal axis of the tibia could not be determined (i.e where tibial metaphysis and diaphysis were not visible). True lateral radiographs were required to measure posterior offset (both femoral condyles being superimposed, giving the appearance of a single femoral condyle). If any images were deemed to be inadequate, all other images belonging to the same patient were excluded from analysis and a replacement patient was found.

### Formulae for the IJLCM technique

Change in joint line height =

Post-operative joint line height (mm)–pre-operative joint line height (mm)

Negative values indicate depression of the joint line height.

### Formulae for the measuring change in posterior offset

Change in posterior offset =

Post-operative posterior offset (mm)–pre-operative posterior offset (mm)

The study adheres to the 1964 Helsinki declaration and its later amendments. Institutional approval was given for the retrospective assessment and analysis of medical records and radiographic images by Imperial College Healthcare NHS Trust (Reference: TRA_183) where all image and statistical analysis was performed. All radiographic images assessed as part of the study were anonymised prior to being sent for analysis at Imperial College Healthcare NHS Trust. Institutional approval was not requested from the other participating units. Institutional approval did not require patient consent for retrospective analysis of anonymised radiographic images.

#### Surgical technique

Conventional jig-based TKA was performed using intra-medullary or extra-medullary cutting guides. Gap balancing, stability and patella tracking was checked initially with trial components prior to final components being implanted.

raTKA was performed using the NAVIO Surgical System. Anatomical landmarks were registered to determine the centre of rotation of the hip and the centre of the knee and ankle. The size and position of the femoral condyles, tibial plateau and tibial slope was measured. Ligamentous laxity in each knee was also assessed. Bone cuts were performed using a surgical saw or burr and were verified. The final leg axis, stability, gap balancing and range of movement was assessed with trial components prior to final components being cemented into position.

#### Statistical analysis

All data were analysed using Statistical Package for Social Sciences version 26 (SPSS, Version 26 IBM Corp, 2019. IBM SPSS Statistics for Windows, Armonk, NY: IBM Corp). All data sets were found to be normally distributed using a Shapiro-Wilk test. The difference (positive values denoting an elevation in joint line height, or an increase in posterior offset) and absolute difference (non-negative values for the calculated change in joint line height and posterior offset) between pre-operative and post-operative joint line height was calculated. Average values, standard deviations and variance were calculated for each observer. Student’s T Test was used to assess for differences between Conventional and raTKA.

## Results

Patient characteristics between groups are shown in Table 1. The inter and intra-observer reliability of the joint line height measurements was found to be >0.92 for pre-operative and post-operative radiographs.

**Table 1:** Patient demographics.

Characteristics	Conventional TKA	raTKA	p-value
**Age (range)**	65.9 (46.3–88.2)	66.75 (47.2–85.5)	0.30
**BMI (range)**	29.7 (18.1–45.2)	30.2 (18.4–44.1)	0.34
**Side (R/L)**	27/33	24/36	0.12
**Sex (M/F)**	31/29	29/32	0.61
**Pre-op anatomical axis**	4.4° valgus	4.8° valgus	0.52

### Change in joint line height

**Fig 4:**
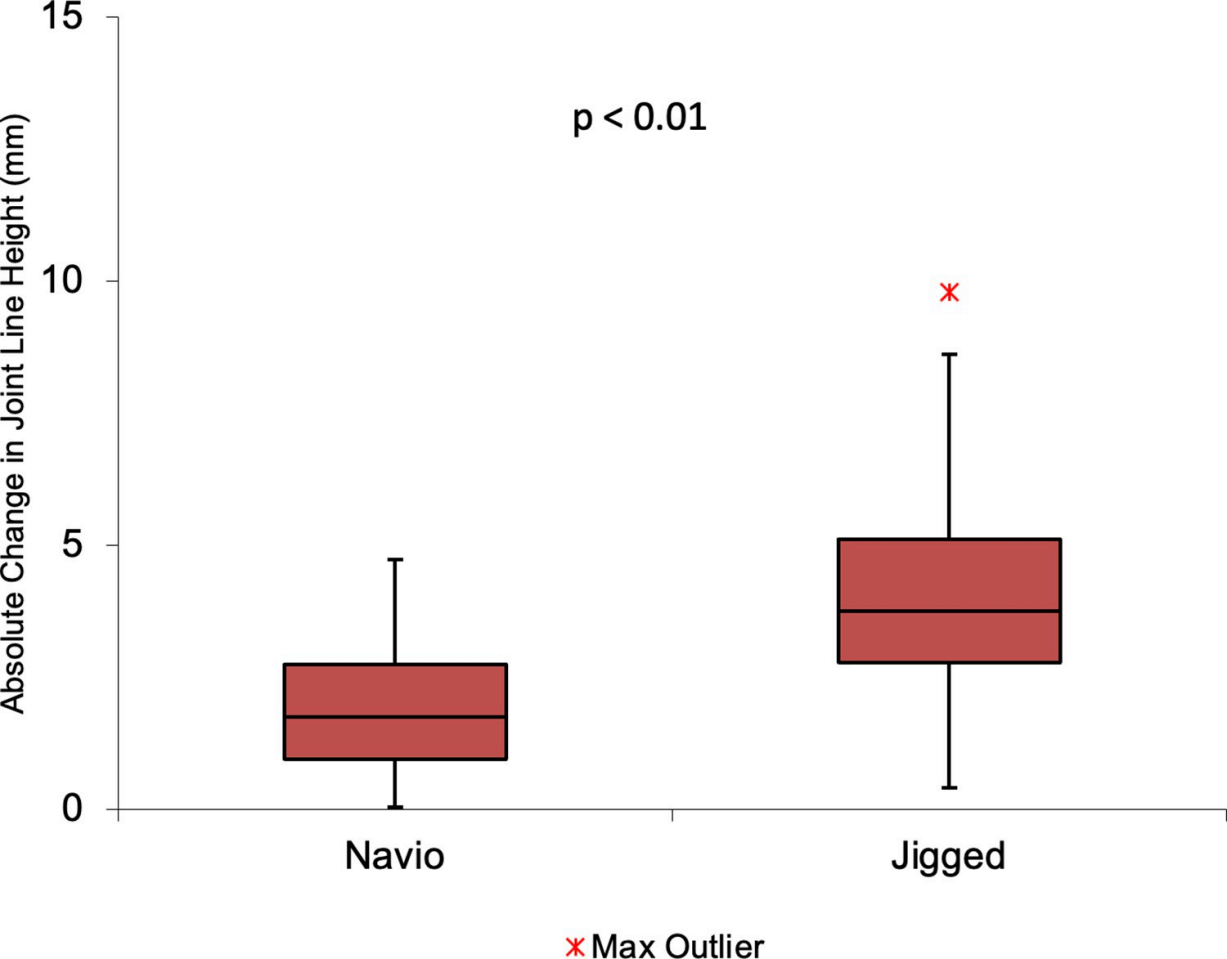
Boxplot for absolute change in joint line height using conventional TKA and Navio TKA.

As shown in Tables 2 and 3, and demonstrated graphically in Fig 4, the absolute difference in joint line height was smaller in the raTKA group. Change in joint line height was <1mm in 17 (28.3%) patients, <2mm in 27 (45.0%) patients and <5mm (100%) in all patients in the raTKA group. Change in joint line height was <1mm in 3 (5.0%) patients, <2mm in 10 (16.7%) patients and <5mm in 43 (71.7%) patients for patients in the conventional TKA group. 17 (28.3%) patients had a change in joint line height greater than 5mm.

**Table 2:** Absolute difference values in change in joint line height between techniques.

	Conventional Technique (mm)	raTKA (mm)
	Absolute Difference	Absolute Difference
**Mean (SD)**	4.00 ± 1.81	1.96 ± 1.21

**Table 3:** Student t test for change in joint line height with raTKA and conventional TKA.

Observer 1	p Value for Absolute Difference
**Measurement a**	p < 0.01
**Measurement b**	p < 0.01
**Combined Measurements**	p < 0.01
**Observer 2**	
**Measurement a**	p < 0.01
**Measurement b**	p < 0.01
**Combined Measurements**	p < 0.01

### Change in posterior offset

**Fig 5:**
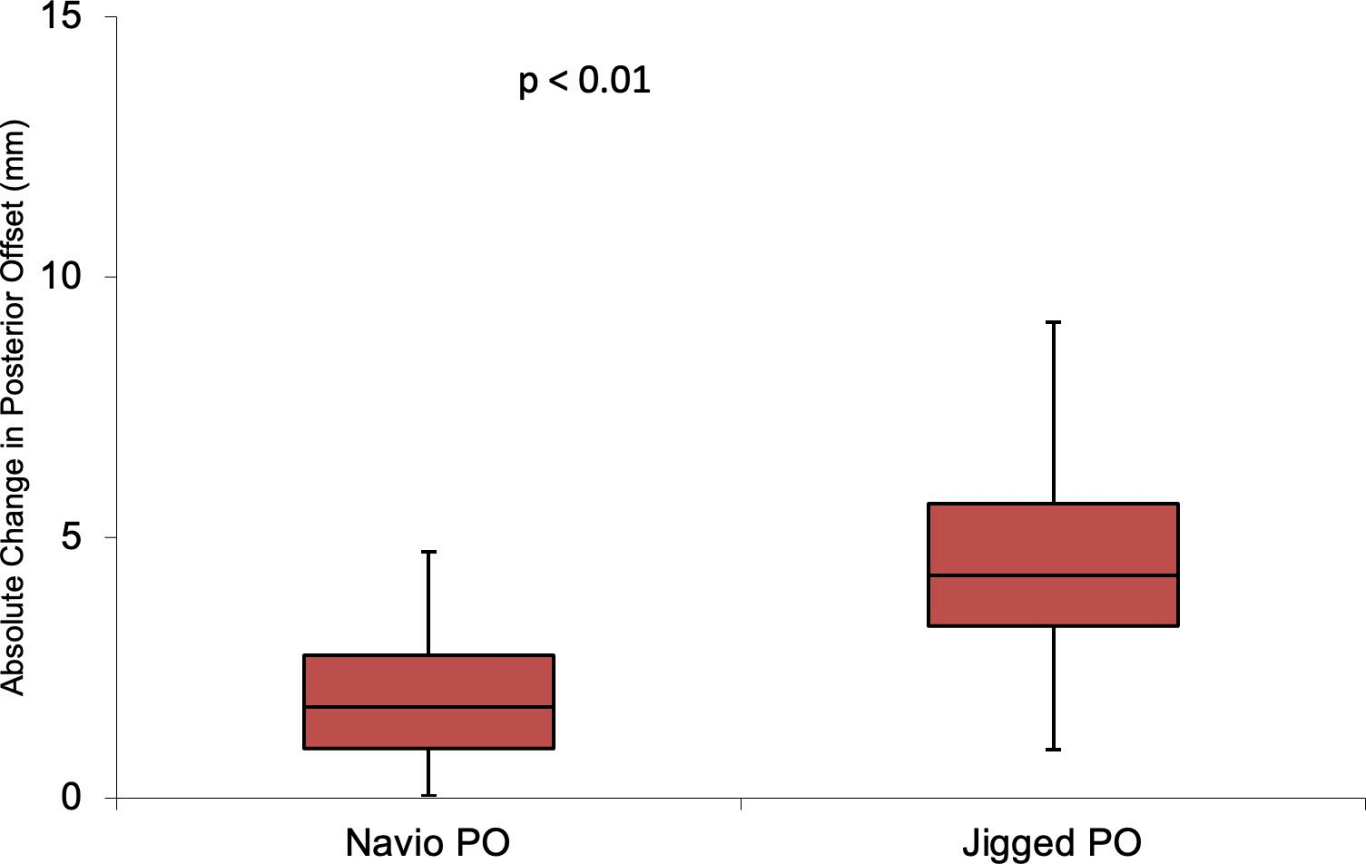
Boxplot for absolute change in posterior offset between conventional TKA and Navio TKA.

As shown in Tables 4 and 5, and demonstrated graphically in Fig 5, the absolute difference in posterior offset was smaller in the raTKA group. Change in posterior offset was <1mm in 22 (35.0%) patients, <2mm in 3 (60.0%) patients and <5mm (100%) in all patients in the raTKA group. Change in posterior offset was <1mm in 4 (6.7%) patients, <2mm in 5 (8.3%) patients and <5mm in 42 patients (70.0%). 18 (30.0%) patients had a change in posterior offset greater than 5mm.

**Table 4:** Absolute difference values for change in posterior offset between techniques.

	Conventional Technique (mm)	raTKA (mm)
	Absolute Difference	Absolute Difference
**Mean (SD)**	4.24 ± 2.52	2.19 ± 1.51

**Table 5:** Student t test for absolute differences for change in posterior offset with raTKA and conventional TKA.

Observer 1	p Value for Absolute Difference
**Measurement a**	p < 0.01
**Measurement b**	p < 0.01
**Combined Measurements**	p < 0.01
**Observer 2**	
**Measurement a**	p < 0.01
**Measurement b**	p < 0.01
**Combined Measurements**	p < 0.01

## Discussion

### Change in joint line height–raTKA vs conventional technique

The most important outcome from this study is that raTKA (using the NAVIO Surgical System) restores the pre-operative joint line in TKA more accurately than when the procedure is performed using a conventional jig-based techniques.

Furthermore, all patients in the raTKA group had their joint line height restored to within 5mm of the pre-operative level. With conventional TKA techniques, joint line height changes were more than 5mm in approximately 30% of patients.

No outcome scores were collected as part of this study. Previous studies have demonstrated a link between joint line height restoration and clinical outcomes. Van Lieshout et al have presented a systematic review demonstrating a statistically significant negative correlation between joint line elevation and post-operative Knee Society Scores (p<0.001) [3]. A deviation of more than 2mm in joint line height has also been shown to have a negative impact on post-operative range of movement [1, 4, 5, 24, 25].

Previous studies have assessed whether raTKA improved joint line height restoration. Jawhar et al used the measurement technique described by Snider & Macdonald [19, 26]. Average change in joint line height was reported as 0.6mm. There was no difference in joint line height restoration between conventional TKA and raTKA. The authors used a measurement tool with an accuracy of 1mm which may lead to inaccuracies in the results presented. Babazadeh et al presented the result of a randomised controlled trial in a smaller cohort than is presented in this study [20]. The authors used an imageless computer navigation system (Ci System, Depuy) and demonstrated no significant difference when change in joint line height when compared with conventional TKA.

Herry et al demonstrated that raUKA enabled more accurate restitution of joint line height in unicompartmental knee replacements (1.4mm ± 2.6mm vs 4.7mm ± 2.4mm (p<0.05) [27]. Kawamura et al presented 73 TKAs with an average joint line elevation of 3.5mm [28], Ritter et al. demonstrated changes of 2.6mm and 2.8mm [29] and Wyss et al. reported on 106 TKAs implants using a soft tissue balancing technique where the average joint line change was 0.3mm [24].

Other authors have attempted to measure joint line height on lateral radiographs. The seminal work by Figgie et al. demonstrated an average change in joint line of 8.9mm following primary conventional TKA. The measuring technique has subsequently been demonstrated to have relatively poor inter and intra-observer reliability [22].

### Change in posterior condylar offset–Navio and conventional technique

This study demonstrates that raTKA enables restoration of posterior condylar offset more accurately than with conventional techniques. Furthermore, all patients in the raTKA group had their posterior condylar offset restored to within 5mm of the pre-operative level. With conventional TKA techniques, posterior condylar offset was more than 5mm different in approximately 30% of patients.

Restoration of the posterior condylar offset contributes to stability and range of movement following TKA. Goutham et al. [30] demonstrated that alteration of PCO by more than 3mm had a negative impact on post-operative range of movement in cruciate-retaining TKA. Change of PCO did not impact ROM in cruciate-sacrificing TKA. Further studies have investigated the impact of change in PCO on ROM with variable results [23, 31–34].

This study had its limitations which included the use of short-leg AP and lateral radiographs. All images were reviewed by both observers for appropriateness. Furthermore, this study did not report patient reported outcomes or functional outcomes. The aim of this paper was to assess how accurately joint line height and posterior condylar offset is restored using different techniques, hence the other outcomes, although important clinically, are not relevant to this study. Due to the different methods used to measure joint line height in the literature, it is difficult to compare our findings with those of other authors. Previous work has demonstrated the accuracy, precision and reliability of the measurement technique utilised in this study [22].

## Conclusion

The NAVIO Surgical System has been shown to help surgeons reproduce the native joint line height and posterior-offset in TKA with greater accuracy than is possible with conventional techniques.

## Supporting information

S1 Data (XLSX)
